# Dense Trajectories and DHOG for Classification of Viewpoints from Echocardiogram Videos

**DOI:** 10.1155/2016/9610192

**Published:** 2016-02-29

**Authors:** Liqin Huang, Xiangyu Zhang, Wei Li

**Affiliations:** School of Physics and Information Engineering, Fuzhou University, Fuzhou 350116, China

## Abstract

In echo-cardiac clinical computer-aided diagnosis, an important step is to automatically classify echocardiography videos from different angles and different regions. We propose a kind of echocardiography video classification algorithm based on the dense trajectory and difference histograms of oriented gradients (DHOG). First, we use the dense grid method to describe feature characteristics in each frame of echocardiography sequence and then track these feature points by applying the dense optical flow. In order to overcome the influence of the rapid and irregular movement of echocardiography videos and get more robust tracking results, we also design a trajectory description algorithm which uses the derivative of the optical flow to obtain the motion trajectory information and associates the different characteristics (e.g., the trajectory shape, DHOG, HOF, and MBH) with embedded structural information of the spatiotemporal pyramid. To avoid “dimension disaster,” we apply Fisher's vector to reduce the dimension of feature description followed by the SVM linear classifier to improve the final classification result. The average accuracy of echocardiography video classification is 77.12% for all eight viewpoints and 100% for three primary viewpoints.

## 1. Introduction

The echocardiography video still plays an important role in modern medical diagnosis. It can be used to analyze the heart by providing the cardiac structural and motional information. The echocardiography video gets the 3D detailed anatomical structure and functional information of the heart from eight standard views, which are usually taken from an ultrasound transducer at the three primary positions (Apical Angles (AA), Parasternal Long Axis (PLA), and Parasternal Short Axis (PSA)). In order to choose the most helpful echocardiography video for disease diagnosis or research, we need to categorize the eight kinds of echocardiography videos. Classifying is the first step in the research. In clinical practice, because of the ultrasound characteristic limits, such as the high noise caused by the low contrast ratio in echocardiography video, sonographer has to manually classify these echocardiography videos. It causes the great decrease of working efficiency and easily impacts the recognition results owing to sonographer experience and image resolution. Therefore, how to use the computer to classify the echocardiography video is a crucial step in the current echo-cardiac research.

In recent years, there are a lot of researchers who focus on this area. For example, Kumar et al. [1] are devoted to expressing the main anatomical structure of space motion. Zhou et al. [2] and Beymer et al. [3] mention echocardiogram classification based on multiple object space relations. Then Shalbaf and Sera et al. [4, 5] add the movement information on this basis, which extract and detect the feature by tracking the movements and patterns of the contour shape of the heart. Wang et al. [6] extract feature by analyzing the factor in video. Guo et al. [7] propose an echocardiography video classification method based on sparse representation and greatly enhance the recognition accuracy. An echocardiography video classification method based on the 3DSIFT feature description is proposed in [8]. The authors detect echo-cardiac features applying cuboid detector, represent these features into 3DSIFT descriptors, and finally use Bag of Word to encode before classifying in SVM. After that, Rap and Espinola-Zavaleta et al. [9, 10] propose the echocardiography classification method based on the features of low dimension and improve the classification result by combining the kernel function with movement decomposition algorithm.

In this paper, it is a challenge to precisely classify the echocardiography video because it has strong noise and low resolution. We propose a method based on the dense trajectory [11] and difference histograms of oriented gradients (DHOG). Dense trajectory tracking, which samples the video points densely in our experiment, can ensure the feature points covered integrally. In addition, it can differentiate the foreground and the background from the scene and improve the efficiency of recognition. Moreover, dense optical flow can improve perfectly the trajectory characteristics of the feature points. In order to describe the dense trajectory, this paper also proposes a method based on motion boundaries and structure descriptors (DHOG, HOF, and MBH), which has the better result. The movement boundary histogram has higher robustness than other descriptions which are based on the optical flow algorithm. It has a great impact for the stability of the classification system. Finally the Fisher vector and linear SVM classifier are applied to classify echocardiography videos.

## 2. Methodology

**Figure 1:**
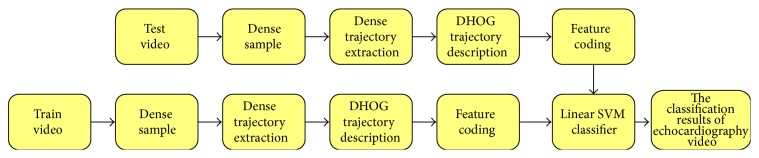
Echocardiography video classification flow based on the dense trajectory.

Dense trajectories are obtained through dense-sampling the points in each frame and track these points in dense optical flow field. Dense sampling ensures the integrity coverage of the feature points of video, and dense optical flow can improve the properties of trajectory, distinguish the foreground and background of echocardiography video, and promote the efficiency of recognition. The flow chart of dense trajectory behavior recognition method is shown in Figure 1. We will produce details about the flow chart in the following content.

### 2.1. Dense Sampling

Feature points on a grid spaced are densely sampled by *W* pixels. Sampling is carried out on each spatial scale separately. It means that feature points equally cover all spatial positions and scales. According to the previous experiment results [7], the sampling step size of *W* = 5 pixels is dense enough to give good results over all data. Smaller sampling step does not have more improvement and it will increase the amount of calculation. There are at most 8 spatial scales in total, depending on the resolution of the video. The spatial scale increases by a factor of $1/\sqrt{2}$.

Because sampling in the homogeneous area makes no sense for detecting, we remove points in these areas using Zhang's criterion [19]. When the eigenvalue of the local autocorrelation matrix is less than a certain threshold, the point will be removed according to the criterion. In this paper, the threshold *T* is as follows:

(1) $$\begin{array}{c} T=0.001\times \underset{i\in I_{t}}{\max}\min\left(\;\lambda_{i}^{1},\lambda_{i}^{2}\;\right), \end{array}$$

where (*λ*_*i*_^1^, *λ*_*i*_^2^) are the eigenvalues of point *i* in the image *I*_*t*_. And experimental results showed that the value of 0.001 represents a good compromise between saliency and density of the sampled points.

### 2.2. Dense Trajectory Extraction

**Figure 2:**
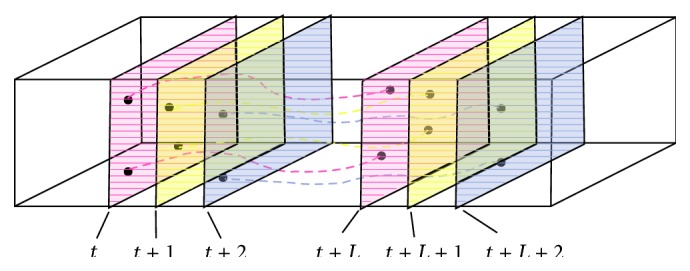
The diagram of dense trajectory extraction.

Dense trajectories are extracted by tracking feature points in each scale space alone, as shown in Figure 2.

For each frame *I*_*t*_, its dense optical flow field *ω*_*t*_ = (*u*_*t*_, *v*_*t*_) is computed by *w*, *r*, *t*. The next frame is where *u*_*t*_ and *v*_*t*_ are the horizontal and vertical components of the optical flow. Given a point *P*_*t*_ = (*x*_*t*_, *y*_*t*_) in frame *I*_*t*_, its tracked position in frame *I*_*t*+1_ is smoothed by applying a median filter:

(2) $$\begin{array}{c} P_{t+1}=\left(\;x_{t+1},y_{t+1}\;\right)=\left(\;x_{t},y_{t}\;\right)+{\left.\;\left(\;M*\omega_{t}\;\right)\;\right|}_{\left(x_{t},y_{t}\right)}, \end{array}$$

where *M* is the median filtering kernel and the size is 3 × 3 pixels. It avoids trajectories of points located on motion boundaries being smoothed out [20].

Once the dense optical flow field is computed, points can be tracked very densely without additional cost. Another advantage using the dense optical flow field is the smoothness constraints which allow relatively robust tracking of fast and irregular motion patterns. In this paper, we use Li's algorithm [21] to embed a translation motion model between neighborhoods of two consecutive frames. And polynomial expansion is employed to approximate pixel intensities in the neighborhood. Not only can this method effectively cooperate with optical flow and improve the accuracy of the optical flow, but also its principle is simple and it has low computational complexity. In our research, we use the implementation from the OpenCV library to complete the calculation of dense optical flow field.

The shape descriptor of a trajectory means encoding local motion positions. Given a trajectory of length *L*, we describe its shape by a sequence (Δ*P*_*t*_,…, Δ*P*_*t*+*L*−1_) of displacement vector Δ*P*_*t*_ = (*P*_*t*+1_ − *P*_*t*_) = (*x*_*t*+1_ − *x*_*t*_, *y*_*t*+1_ − *y*_*t*_). The resulting vector is normalized by the sum of displacement vector magnitudes:

(3) $$\begin{array}{c} T=\frac{\left(\Delta P_{t},\ldots ,\Delta P_{t+L-1}\right)}{\sum_{j=t}^{t+L-1}\left\|\Delta P_{j}\right\|}. \end{array}$$

In the following, we refer to this vector as trajectory. As we use trajectories with a fixed length of *L* = 15 frames, we obtain a 30-dimensional descriptor.

### 2.3. Motion and Structure Descriptors

Besides the trajectory shape information, we also design descriptors to embed appearance and motion information and include difference histograms of oriented gradients (DHOG), histograms of optical flow (HOF), and the motion boundary histograms (MBH). These features are combined as a motion model of the local feature descriptor. In *L* frames of continuous video, we extract the feature in the *N* × *N* pixels of local area. In order to embed structural information, we divide the *N* × *N* × *L* spatiotemporal cube into a set of grids with *n*_*σ*_ × *n*_*σ*_ × *n*_*τ*_ and compute the local descriptor corresponding to each grid. Finally, we combine each descriptor into the final vector.

#### 2.3.1. Different Histograms of Oriented Gradients

HOG feature [22] is the information description based on the local statistical characteristics; its main thought is that the local target appearance and shape in an image can be described by the gradient or the direction of the edge density distribution. First the image is divided into several nonoverlapping cells with size *N* × *N*, and the gradient of each cell is calculated and then represented using histogram statistics. By merging multiple cells into a vector, the HOG features are obtained.

**Figure 3:**
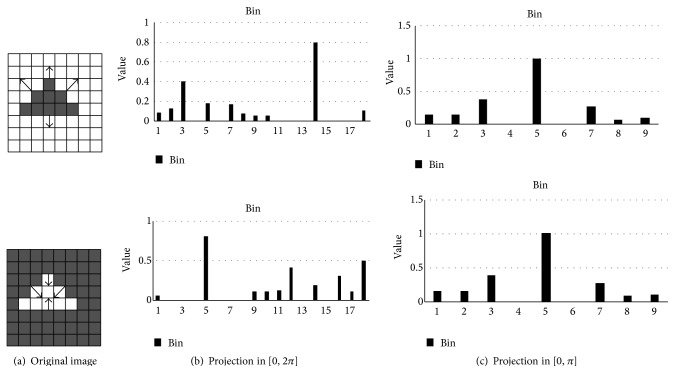
The robustness of the compression direction on background.

Although the traditional HOG features have strong robustness for certain amount of illumination change, the expression ability of HOG feature still exists. Since the gradient direction can be changed by the light and shade of the background, it is possible to generate different expressions for the HOG feature as shown in Figure 3. The projection in the interval [0, *π*] has no change for the gradient direction histogram, as shown in Figure 3(c).

It can lead to ignoring the difference information of some target showed. The HOG feature descriptor is uniform because of the different shape objects with different opposite direction of gradient and the same size and number, which can produce the same histogram. So the HOG features have no distinguished ability for these objects.

**Figure 4:**
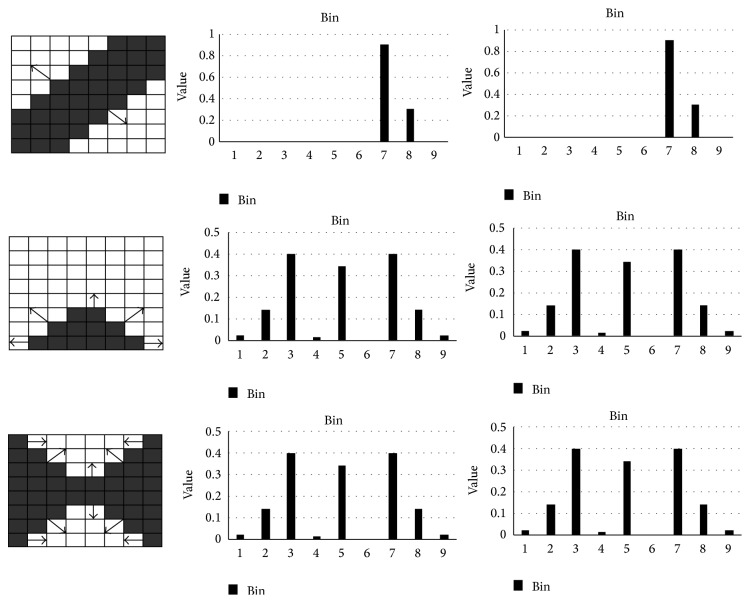
Comparison of HOG and DHOG.

In order to improve the expression ability of HOG feature in echocardiography video characteristic trajectory. We propose an improved HOG descriptor algorithm. We divide *L* into [0, 2*π*] area and get the gradient direction histogram *H*_*g*_, where *L* is even and each pixel in the cell needs to be vote:

(4) $$\begin{array}{c} H_{og}\left(\;i\;\right)=H_{g}\left(\;i\;\right)+H_{g}\left(\;i+\frac{L}{2}\;\right),\;1\leq i\leq \frac{L}{2}, \end{array}$$

where *H*_*og*_(*i*) and *H*_*g*_(*i*) are, respectively, *i*th element values of HOG and *H*_*g*_. In this paper, we set a new series of histograms *H*_*ng*_ whose length is equal to the HOG after the HOG, as shown in Figure 4, and each element value is

(5) $$\begin{array}{c} H_{ng}\left(\;i\;\right)=\left|\;H_{g}\left(\;i\;\right)-H_{g}\left(\;i+\frac{L}{2}\;\right)\;\right|,\;1\leq i\leq \frac{L}{2}. \end{array}$$

In this paper, we call the new gradient direction histogram as difference HOG (DHOG); in order to simplify the calculation, we extract the HOG feature descriptors in 32 × 32 pixels of dense trajectory. Each block is divided into 2 × 2 cells. In order to obtain the optimal description while considering the differences in the opposite direction in this paper, the area (0~180°) is divided into nine bins and then extended to [0, 2*π*] by DHOG. We divide the gradient direction area of 0~360° into 18 bins, and calculate gradient direction histogram in each cell. Then we divide the *L* = 15 frames' dense trajectory into three parts and sum the DHOG features with the corresponding bin. The eigenvectors also are normalized with the *L*2-norm. So, in this paper, there is a dense trajectory of HOG feature with *L* = 15 frames, whose dimensionality is 2 × 2 × 18 × 3 = 216.

#### 2.3.2. Gradient and Optical Flow Histograms

Different from the DHOG descriptor extracting the static shape information, the HOF uses optical flow to describe and extract the motional information. The calculation method of HOF is the same as DHOG. HOF makes the sum of modulus value of light flow's horizontal component and vertical component as the amplitude of the light flow and makes the arctangent value of the ratio of vertical and horizontal component as the direction angle of the light flow. Then the light flow direction will be projected into the adjacent bin which is based on the amplitude of light flow. Considering that the light flow amplitude is small (less than a certain threshold), we will add a zero bin in the HOF feature which is used to hold the number of pixels. HOF descriptor also is normalized with the *L*2-norm. The other parameters are the same as the DHOG. So the dimensionality of HOF is 2 × 2 × 9 × 3 = 108.

#### 2.3.3. Motion Boundary Histograms

Motion boundary histograms (MBH) are proposed by Yao at el. for detection of the human in 2014 [23]. It computes derivatives separately for the horizontal and vertical components. MBH is the gradient of light flow; it can get rid of uniform motion and keep the change of optical flow field. In a certain extent, it is a complement to the DHOG and HOF that is removing the camera movement to the human. The DHOG and HOF are generated from the optical flow, which makes the computational complexity of MBH small.

### 2.4. The Selection of Feature Encoding and Classifier

In this paper, we use the Fisher vector [24, 25] with the improved 256 visual words of GMM (signed square-rooting followed by *L*2-norm) for features encoding. Fisher's vector seen as the expansion of the BOV word bag model is the intermediate expression of image. Because Fisher's vector fuses the advantages of production and is discriminant of Fisher's kernel architecture, it can not only reflect the frequency of each visual word but also encode the differences of local feature in visual words information. In addition, it can represent the richer image feature compared to the word bag model. The high dimensional feature that combined with simple and effective linear classifier can achieve good classification effect. So, in this paper, we use the linear SVM classifier, which has the excellent performance of machine learning in classification and generalization. So linear SVM classifier has a lot of advantages compared to other learning algorithms in practice and is simple and convenient to use. Its performance is better in classification.

## 3. Experimental Results and Analysis

### 3.1. Data

In this experiment, 228 different echocardiography videos are collected from 72 different patients (including 58 normal individuals and 14 patients with cardiac wall motion abnormalities); these data were provided by Tsinghua University First Hospital. All the length of echocardiography video is 1 to 2 seconds and the videos are stored as 434 × 636 resolution and in 26-frame DICOM format (Digital Imaging and Communications in Medicine).  Table 1 shows the detailed number of eight kinds of video.

### 3.2. Experiment Setup and Result Analysis

In this experiment the dense sampling grid of feature points is selected with 5 pixels in the process of dense sampling, and it can guarantee the best result. Then the tracking frame length is set to 15 frames in the process of tracking of feature points. In the process of motion boundary description, the default parameters are *N* = 32, *n*_*σ*_ = 2, and *n*_*τ*_ = 3 in our experiment, and these parameters have the best effect through the experiments testing. In terms of selecting the feature encoding and classifier, this experiment adopts vlfeat-0.9.19 software for processing (download from http://www.vlfeat.org/). For the selection of training video and testing video, we use the method of “half and half” (the total echocardiography video data are randomly selected, half as the training video and the other half as the testing video). And we analyze its classified results.

We use the confusion matrix to show the classification accuracy rate of the eight kinds of echocardiography video in Table 2, and the calculation method of accuracy rate is showed in function 3.1. Overall, the average classification accuracy of all the classes is 77.12%. We can find that the classification accuracy for each class is different. In particular, the classification accuracy for these classes, such as A5C, PSAB, and PSAP, is lower than the others. The main reason is shortness of experiment data corresponding to these three kinds of echocardiography videos. It leads to lack of training samples:

(6) $$\begin{array}{c} accuracy\;\;rate=\frac{number\;\;of\;\;correct\;\;videos}{number\;\;of\;\;videos}. \end{array}$$

In view of the above experimental result that the classification accuracy can be affected by the number of the sample videos, in the following experiment, we cut off the less experimental data of three kinds of video (A5C, PSAB, and PSAP) and make further experiment with the rest of the five kinds of experimental data and compare the results (Table 3).

From Table 3, comparing with the eight kinds of echocardiography video and five kinds of the echocardiography video, we can find that the classification accuracy shows a significant improvement. The classification accuracy is promoted from original 77.12% to 98.36%.

Our experiments also make a classification test for echocardiography video of the primal three kinds of locations (AA, PLA, and PSA), its average classification accuracy is 100%, and it can satisfy the basic needs of medical echocardiography video classification.

Finally, compared with the other echocardiography video classification algorithm which is previously proposed our experimental result is increased a lot (illustrated in Table 4). And for the echocardiography video of the three kinds of primal locations, our experiment has the best accuracy. Therefore, the method of echocardiography video's classification based on dense trajectory tracking and DHOG has an obvious improvement in accuracy compared with the previous algorithm.

## 4. Summary and Discussion

This paper is based on the classification of the echocardiography video with the dense trajectory tracking and DHOG algorithm. Firstly, we use the dense trajectory tracking and DHOG algorithm to describe the feature in each kind of echocardiography video and get the features and then apply the VLfeat software for Fisher vector to encode the features. Finally we use the linear SVM to get the classification results. From the experimental results, while the method adopted in this paper is compared with the previous method which is based on the 3D-SIFT echocardiography video classification, the classification accuracy is improved significantly. But the final average classification accuracy is still not high because of the lack of the echocardiography video data. So, in this paper, we get rid of the small amount of three kinds of experimental data and make the classification experiment again. After solving the problem of the sample data, the average classification accuracy of the rest of five classes of data compared to the average classification accuracy of eight classes has increased nearly 20.9%. And, in three classes of primal position of echocardiography video, the average classification accuracy is 100%. Therefore, the method of this paper can preliminarily achieve the requirement of the medical echocardiography video classification accuracy. For dealing with the classification of echocardiography video, the challenge is not just about the video noise caused by the low resolution of the video but also how to balance the computational complexity and time cost. In the future work, we will gradually increase the database of eight kinds of echocardiography video and adopt or propose newer and more effective method to improve the classification accuracy and reduce the required time of the classification as well.

## Figures and Tables

**Table 1:** The number of each type of video.

Video	A2C	A3C	A4C	A5C	PLA	PSAB	PSAM	PSAP	Sum
Number	45	31	36	7	42	11	39	17	228
Patients	9	6	7	1	8	2	8	4	45
Normal	36	25	29	6	34	9	31	13	183

**Table 2:** Confusion matrix for eight kinds of echocardiography video classification.

	Classified result									Accuracy
		A2C	A3C	A4C	A5C	PLA	PSAB	PSAM	PSAP	
Class	A2C	22	1	0	0	0	0	0	0	0.96
	A3C	1	15	0	0	0	0	0	0	0.95
	A4C	0	0	17	1	0	0	0	0	0.95
	A5C	2	0	1	0	0	0	0	0	0.00
	PLA	0	0	0	0	21	0	0	0	1.00
	PSAB	0	0	0	0	1	3	0	2	0.50
	PSAM	0	0	0	0	0	0	20	0	1.00
	PSAP	0	0	0	0	2	0	0	7	0.81

**Table 3:** Confusion matrix for five kinds of echocardiography video classification.

	Classified result						Accuracy
		A2C	A3C	A4C	PLA	PSAM	
Class	A2C	22	1	0	0	0	0.96
	A3C	1	15	0	0	0	0.95
	A4C	0	0	18	0	0	1.00
	PLA	0	0	0	21	0	1.00
	PSAM	0	0	0	0	20	1.00

**Table 4:** The algorithm accuracy compared with other algorithms in this paper.

The comparison of result		
	Classification accuracy of eight classes	Classification accuracy of three classes
[8]	72%	90%
[12]	70.4%	86%
[13]	74%	91%
[14]	76%	93%
[15]	76.5%	93%
[16]	75%	92%
[17]	73.2%	95%
[18]	71.3%	94%
Our method	77.12%	100%
